# Tibial Lateral Condyle Fracture After Cementless Oxford Unicompartmental Knee Arthroplasty (UKA): A Report of Four Cases

**DOI:** 10.7759/cureus.53228

**Published:** 2024-01-30

**Authors:** Atsuki Tanaka, Takafumi Hiranaka, Takaaki Fujishiro, Motoki Koide, Koji Okamoto

**Affiliations:** 1 Orthopaedic Surgery and Joint Surgery Centre, Takatsuki General Hospital, Takatsuki, JPN

**Keywords:** cementless, pinhole, magnetic resonance imaging (mri), lateral tibial fracture, unicompartmental knee arthroplasty (uka)

## Abstract

Cementless unicompartmental knee arthroplasty (UKA) has a lower rate of radiolucency in postoperative follow-up than cemented UKA. However, the rate of tibial plateau fracture, one of the complications, has been reported to be higher in cementless UKA than in cemented UKA. We report four cases of postoperative tibial lateral condyle fractures after cementless Oxford UKA. Four patients underwent cementless Oxford UKA. Immediate postoperative radiography and CT showed no fracture lines. At five to six weeks postoperatively, MRI showed a fracture line from the intersection of the longitudinal and transverse tibial osteotomies through the lateral pinhole to the end of the lateral tibial diaphysis. At three months, bone union was observed without surgical treatments. Lateral tibial fracture after cementless Oxford UKA has a good clinical course without the need for surgical intervention. Medial fractures should thus be more actively prevented. MRI is useful for less symptomatic tibial lateral condyle fractures.

## Introduction

Unicompartmental knee arthroplasty (UKA) is a highly successful treatment for refractory unicompartmental osteoarthritis [1]. UKA is conducted by a minimally invasive technique compared with total knee arthroplasty (TKA). Advantages, therefore, include shorter postoperative stays, lower postoperative bleeding, and fewer transfusions [2]. Moreover, in a long-term cohort study of the medial UKA, Liddle et al. reported a 10-year survival rate, which set the endpoint for revision surgery at over 90% [3].

However, according to another report based in the United Kingdom, the revision rate of UKA is higher than that of TKA [4]. The common reasons for revision include aseptic loosening and pain [4]. A cementless UKA was introduced to improve these high revision rates; it has a lower rate of radiolucency in the postoperative follow-up than cemented UKA [5]. Conversely, the rate of tibial plateau fracture, one of the complications, has been reported to be higher in cementless UKA than in cemented UKA [3]. These fractures are attributed to the surgical technique and patient background and are mostly medial tibial plateau fractures [6]. Postoperative medial tibial plateau fractures are serious complications in cementless UKA, and surgical treatment is often required.

There have been no previous reports of postoperative tibial lateral condyle fractures; however, there have been reports of medial tibial plateau fractures originating from the tibial cutting guide holding pinholes [7]. Such fractures are thought to be caused by stress concentration in the lower area of the tibial base plate, and stress concentration tends to occur around pinholes [8]. We report four cases of tibial lateral condyle fractures passing through the lateral pinhole of the tibial cutting guide after medial UKA.

## Case presentation

Procedures of Surgery

All patients had knee osteoarthritis resistant to conservative treatment and underwent UKA by the same experienced surgeon. All provided written informed consent to inclusion in this report. Cementless Oxford UKA was used in all cases, with Microplasty® (Zimmer Biomet, Warsaw, IN) instrumentation. The tibial cutting guide, which aims for a 7° posterior slope, and neutral varus/valgus relative to the mechanical tibial axis were used. The tibial cutting guide was fixed in contact with the tibia with headpins. To fix the tibial cutting guide, only one pinhole located above the shaft (lateral pinhole) was used. No fractures were recognized intraoperatively. Beginning on the day after surgery, the patient underwent a full-load rehabilitation.

Case 1

**Figure 1:**
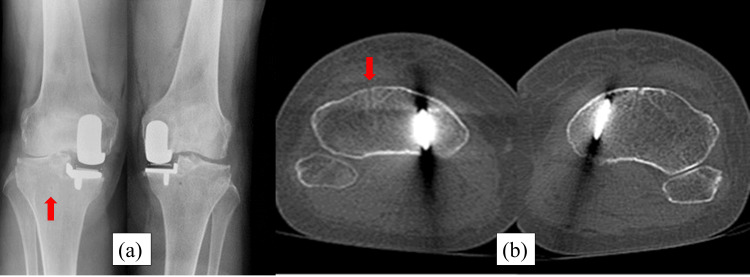
Immediately postoperative radiography and computed tomography (CT). (a) Postoperative radiography shows no fracture line. (b) Postoperative CT shows the level of a pinhole. The red arrows show the place of the pin. The pin was placed outside the tibial mechanical axis.

**Figure 2:**
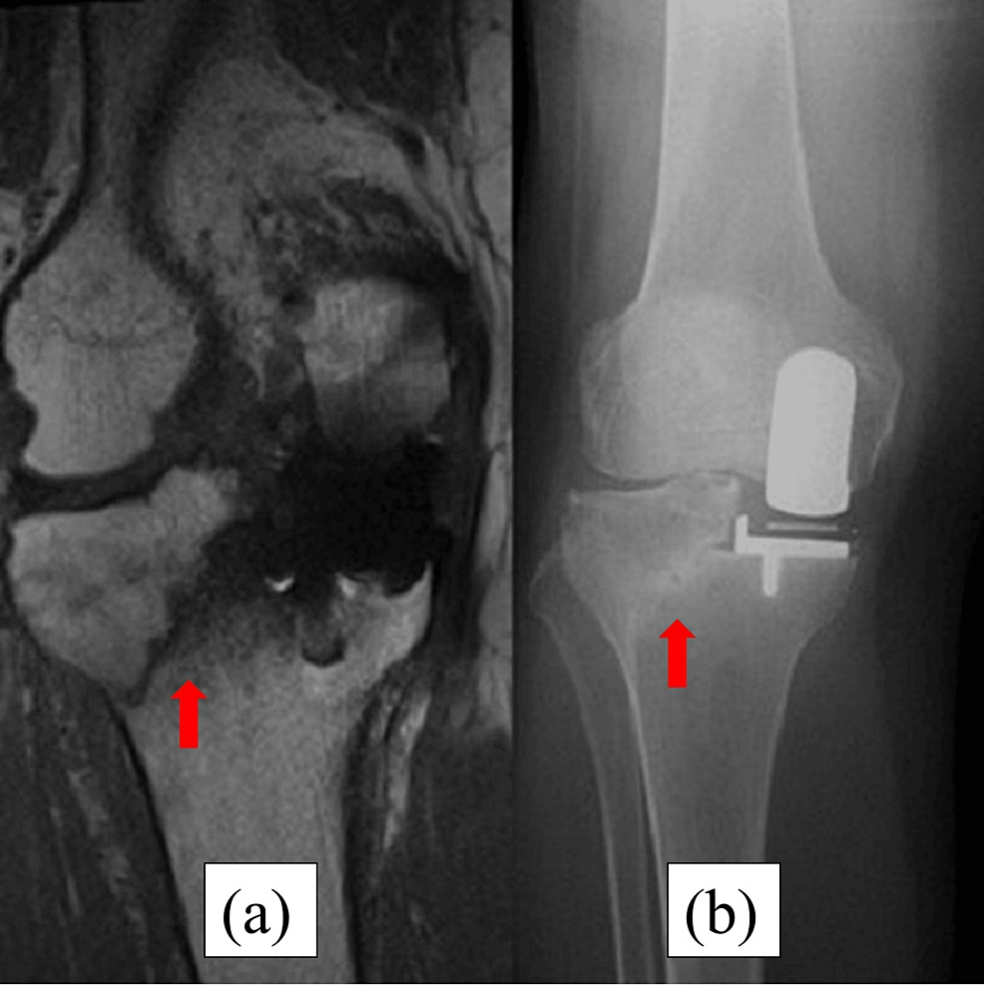
Magnetic resonance imaging (MRI) and radiography at five weeks postoperatively. (a) The red arrow of the MRI shows a fracture line from the intersection of the longitudinal and transverse tibial osteotomies through the lateral pinhole to the end of the lateral tibial diaphysis of the right knee. (b) The red arrow of radiography shows the same fracture line as MRI.

**Figure 3:**
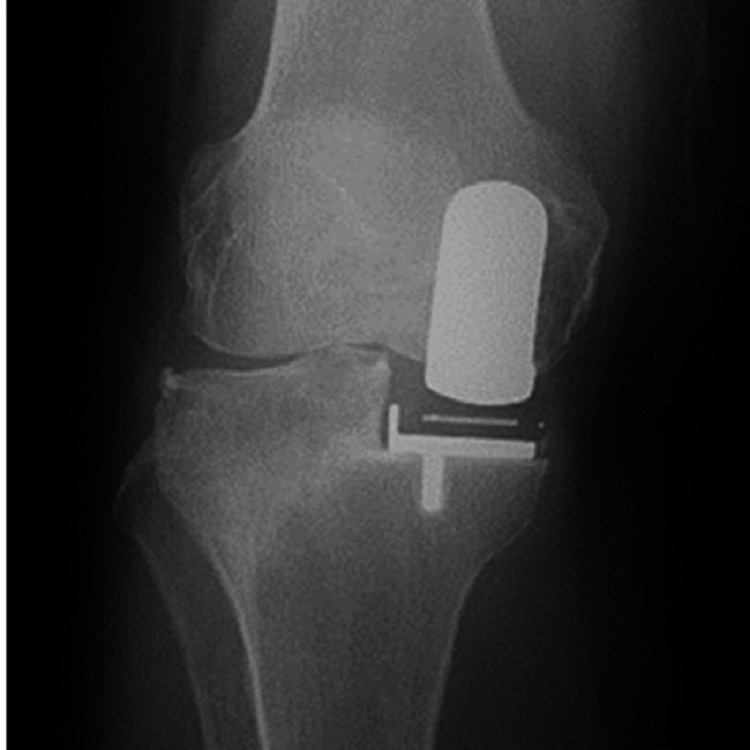
Radiography at three months postoperatively.

**Figure 4:**
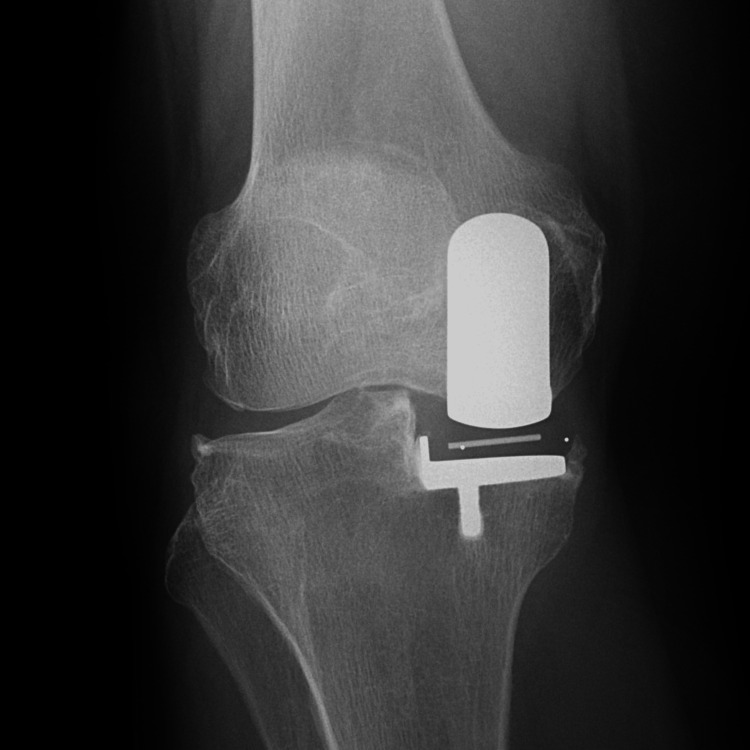
Radiography at two years postoperatively.

An 80-year-old woman (height of 153 cm, body weight of 50 kg, BMI of 21.4 kg/m²) underwent simultaneous bilateral cementless Oxford UKA. She had received conservative treatment for approximately 20 years. Radiography showed the disappearance of both knee medial joint spaces, and she reported severe knee pain in the period before surgery. Immediate postoperative radiography and CT showed no fracture lines (Figure 1). The pin was placed outside the tibial mechanical axis. At two weeks, she could flex both knees sufficiently and was discharged without the need for crutches. At six weeks, she had no pain, tenderness, or swelling around the proximal tibia. However, an MRI showed on the right knee a fracture line from the intersection of the longitudinal and transverse tibial osteotomies through the lateral pinhole to the end of the lateral tibial diaphysis of the right knee (Figure 2). No external fixation, such as a cast or splint, or load limitation was deemed necessary. At three months, bone union was observed (Figure 3). At two years postoperatively, there was no dislocation or loosening of components (Figure 4).

Case 2

**Figure 5:**
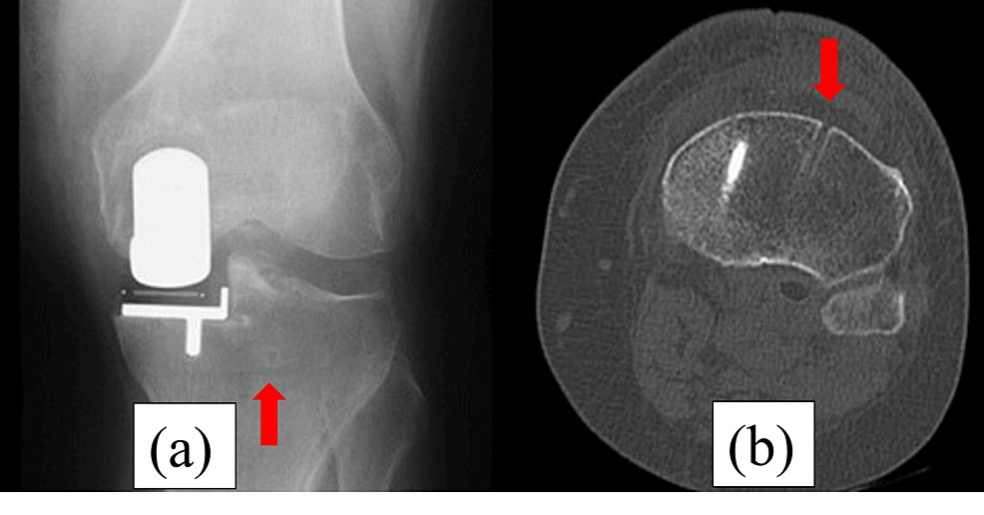
Immediately postoperative radiography and CT. (a) Postoperative radiography shows no fracture line. (b) Postoperative CT shows the level of a pinhole. The red arrows show the place of the pin. The pin was placed outside the tibial mechanical axis.

**Figure 6:**
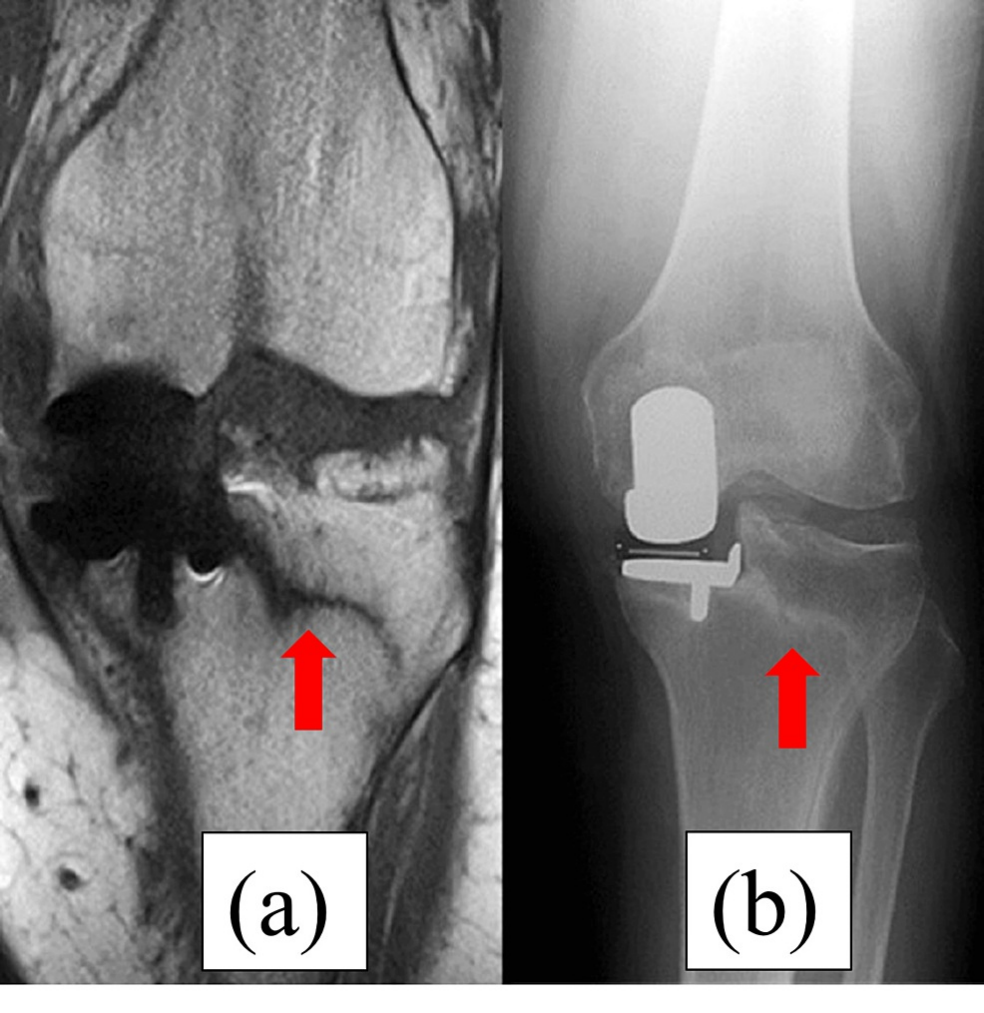
MRI and radiography at five weeks postoperatively. (a) The red arrow of the MRI shows the same fracture line as Case 1. (b) The red arrow of radiography shows the same fracture line as the MRI.

**Figure 7:**
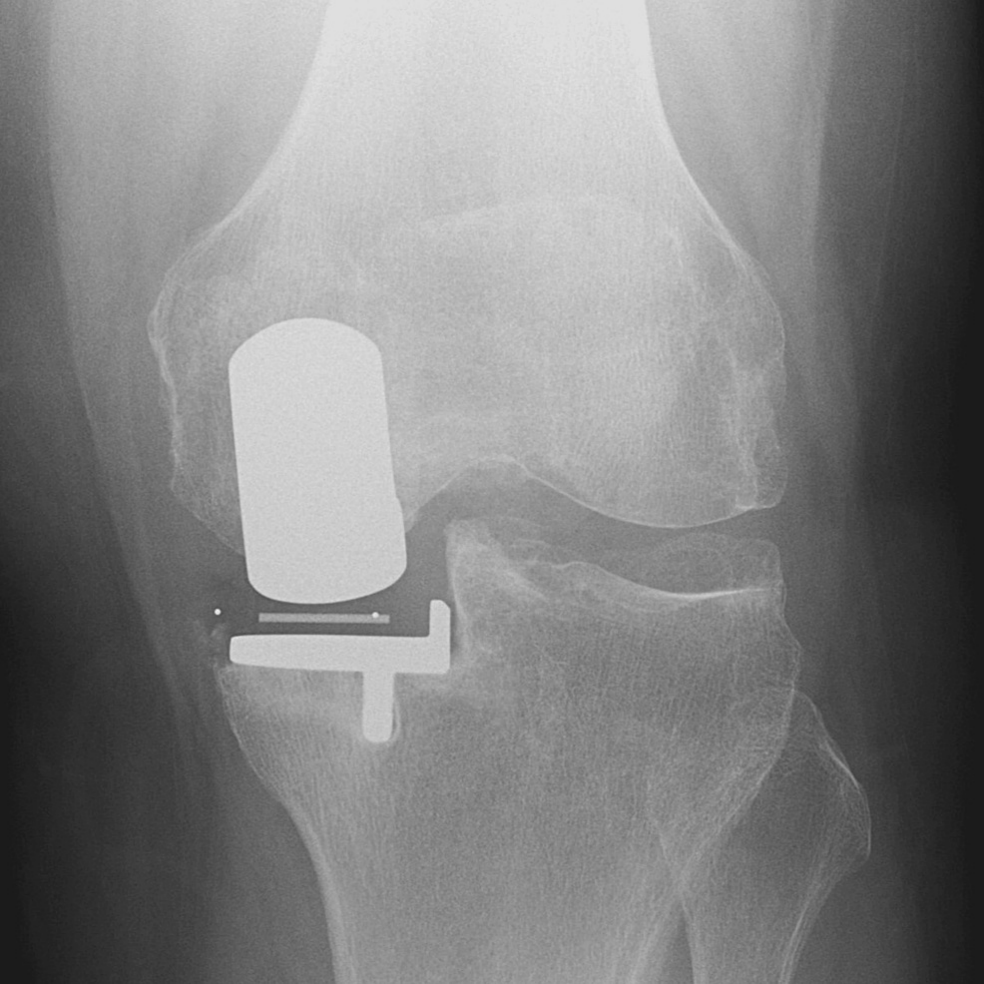
Radiography at three months postoperatively.

**Figure 8:**
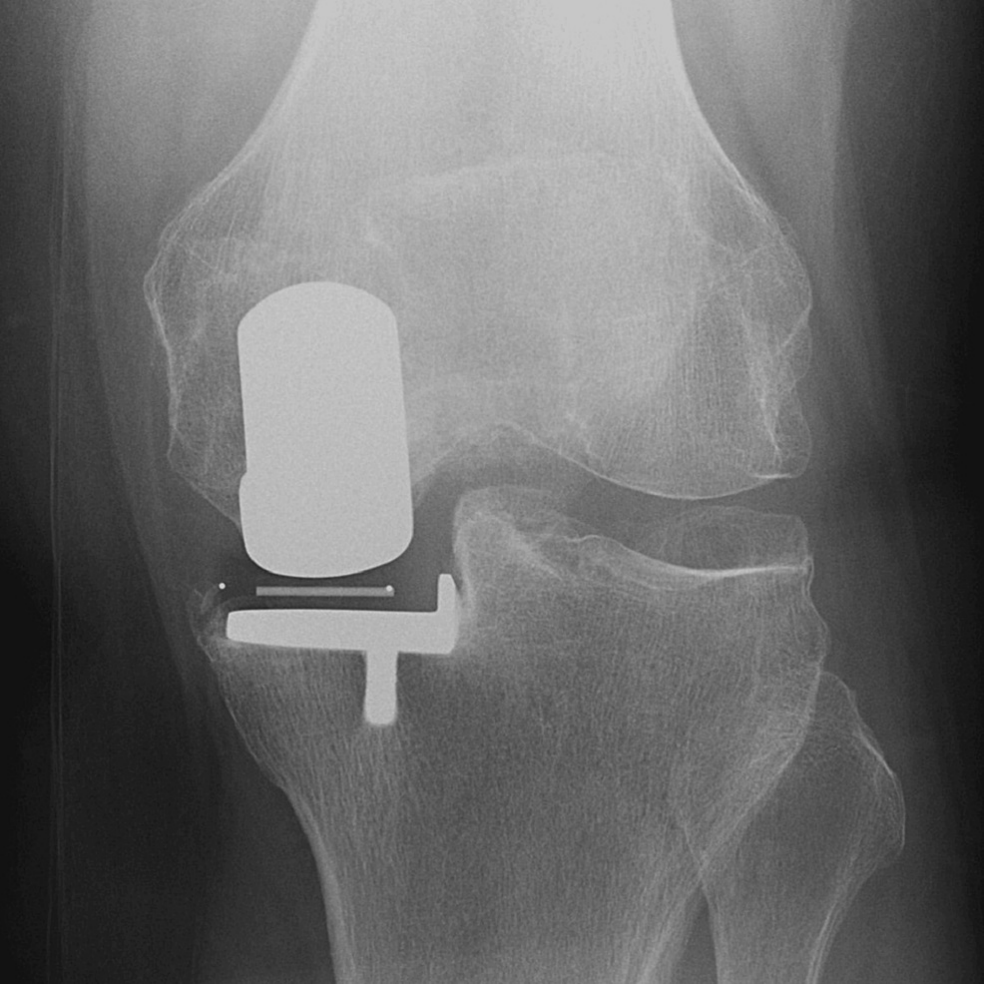
Radiography at two years postoperatively.

A 72-year-old woman (height of 165 cm, body weight of 78 kg, BMI of 28.7 kg/m²) underwent cementless Oxford UKA on the left side because she had severe knee osteoarthritis. Immediate postoperative radiography and CT showed no fracture lines (Figure 5). The pin was placed outside of the tibial mechanical axis. At two weeks, the patient was discharged without the need for crutches. At six weeks, there was no pain, tenderness, or swelling around the proximal tibia; however, an MRI showed a tibial lateral condyle fracture line (Figure 6). No external fixation was performed, and bone fusion was observed at three months (Figure 7). There was no dislocation or loosening of components at two years postoperatively (Figure 8).

Case 3

**Figure 9:**
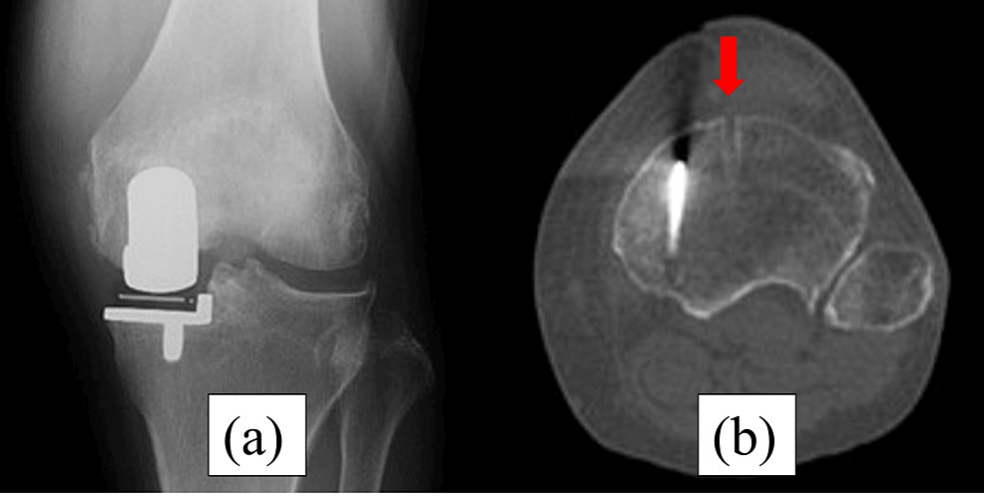
Immediately postoperative radiography and CT. (a) Postoperative radiography shows no fracture line. (b) Postoperative CT shows the level of a pinhole. The red arrows show the place of the pin. The pin was placed on the tibial mechanical axis.

**Figure 10:**
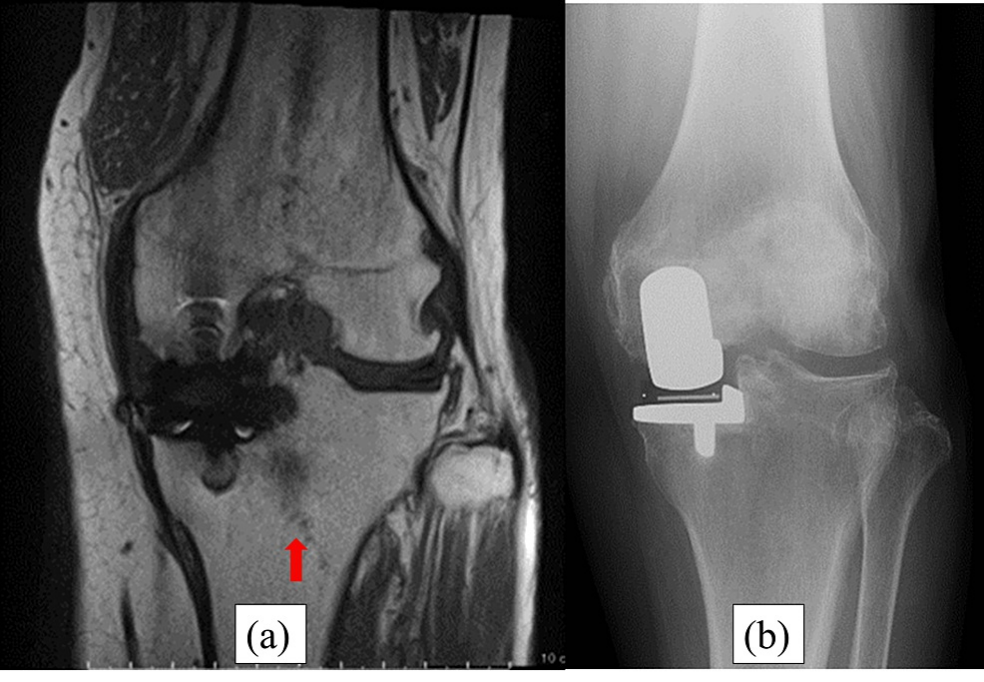
MRI and radiography at five weeks postoperatively. (a) The red arrow of the MRI shows the same fracture line as Cases 1 and 2. (b) Radiography does not reveal a fracture line.

**Figure 11:**
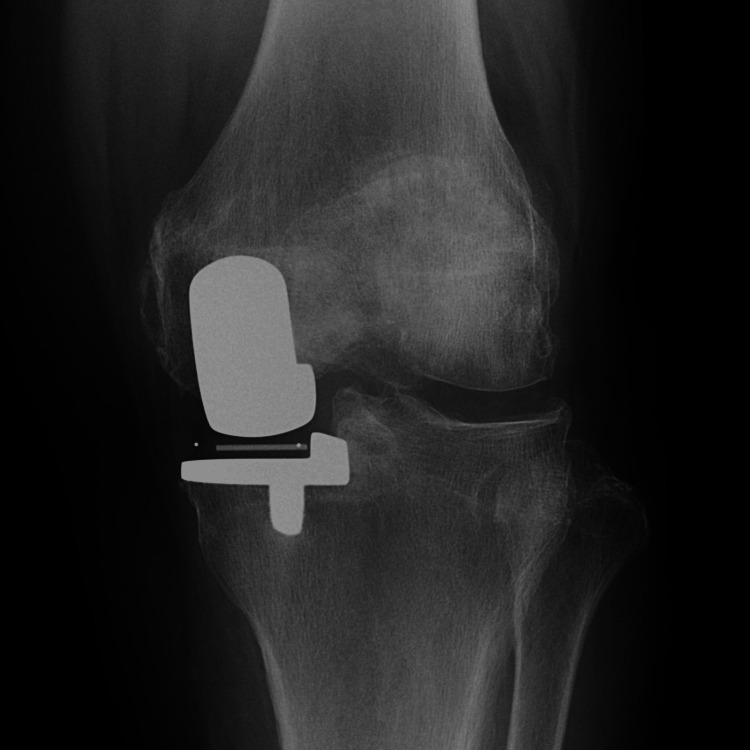
Radiography at three months postoperatively.

**Figure 12:**
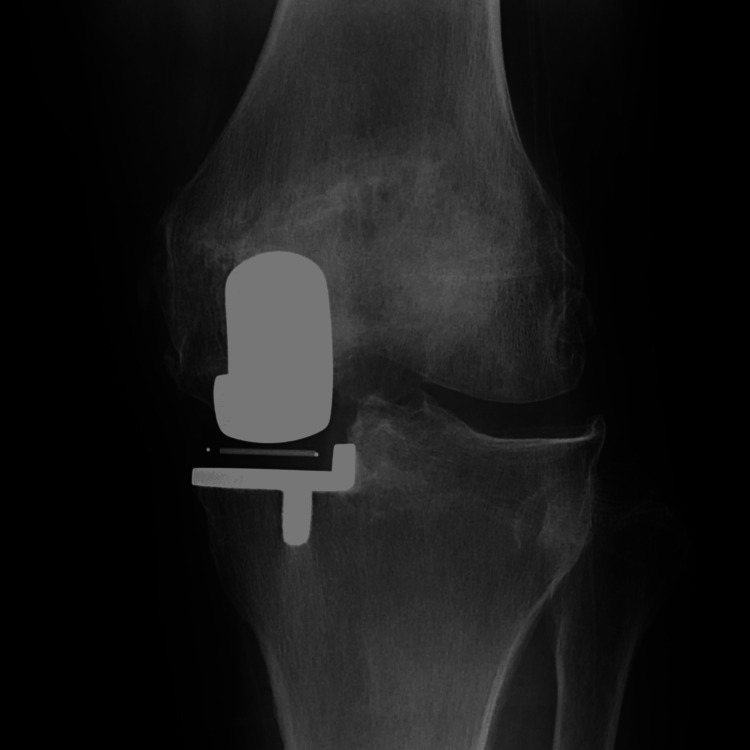
Radiography at two years postoperatively.

A 65-year-old woman (height of 165 cm, body weight of 59 kg, BMI of 21.4 kg/m²) was referred because of a history of severe knee pain. She had received conservative treatment for severe knee osteoarthritis for approximately 12 years. Cementless Oxford UKA on the left side and TKA on the right side were performed simultaneously. Immediate postoperative radiography and CT showed no fracture lines (Figure 9). The pin was placed on the tibial mechanical axis. She was discharged two weeks later without crutches. Six weeks postoperatively, there was no pain, tenderness, or swelling around the proximal tibia. However, as reported in the other cases, MRI showed a fracture line (Figure 10). No external fixation was performed, and bone union was observed three months after surgery (Figure 11). Two years postoperatively, there was no dislocation or loosening of components (Figure 12).

Case 4

**Figure 13:**
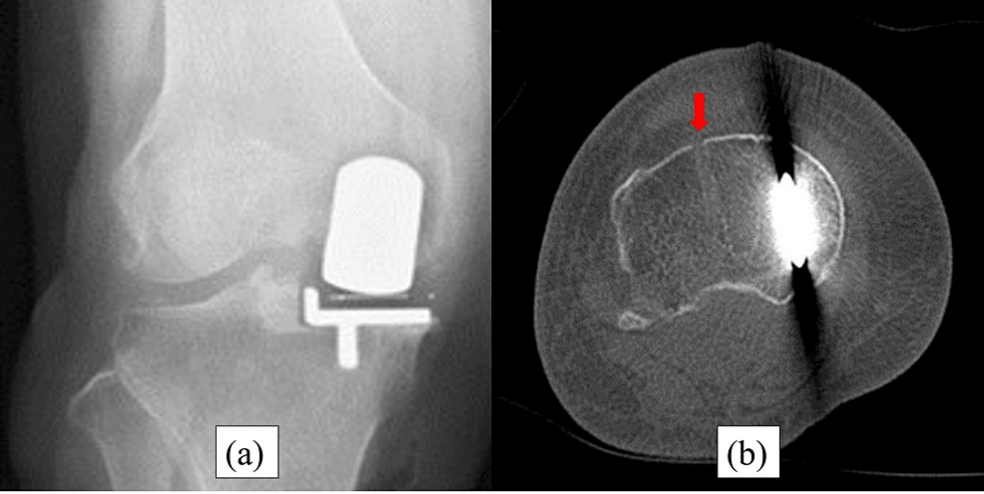
Immediately postoperative radiography and CT. (a) Postoperative radiography shows no fracture line. (b) Postoperative CT shows the level of a pinhole. The red arrows show the place of the pin. The pin was placed on the tibial mechanical axis.

**Figure 14:**
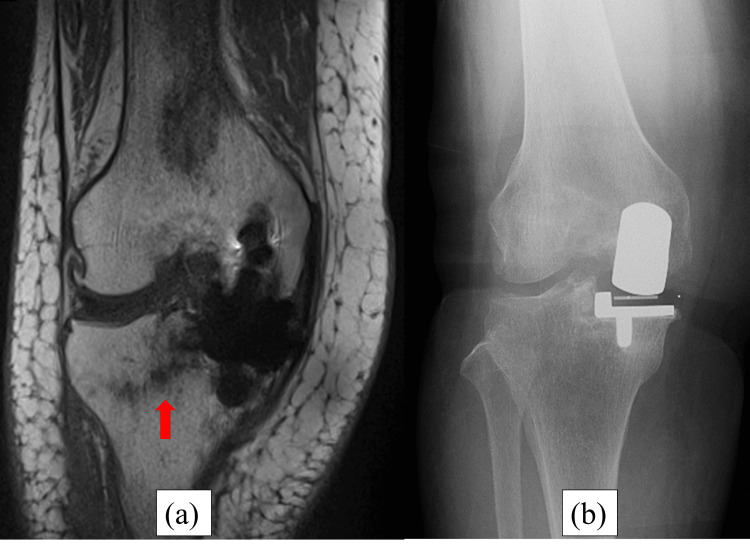
MRI and radiography at five weeks postoperatively. (a) Postoperative radiography shows no fracture line. (b) Postoperative CT shows the level of a pinhole. The red arrows show the place of the pin. The pin was placed on the tibial mechanical axis.

**Figure 15:**
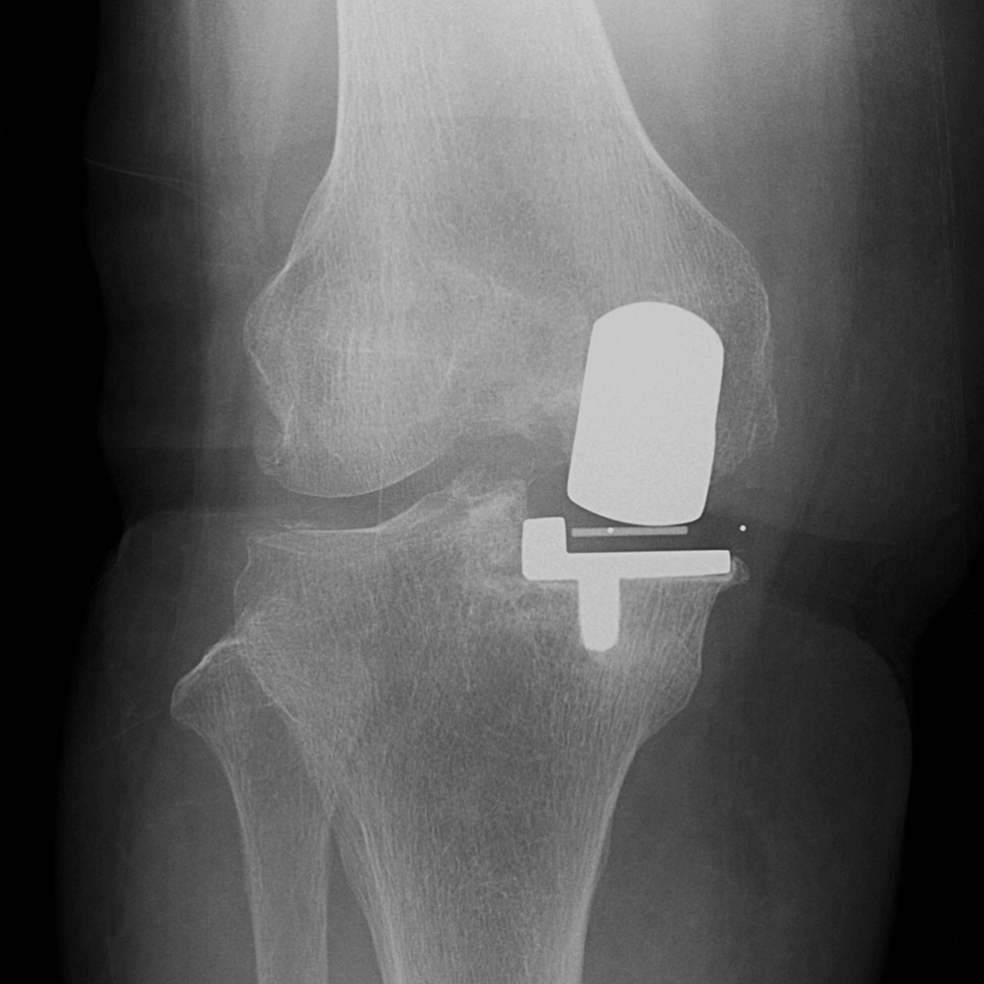
Radiography at three months postoperatively.

**Figure 16:**
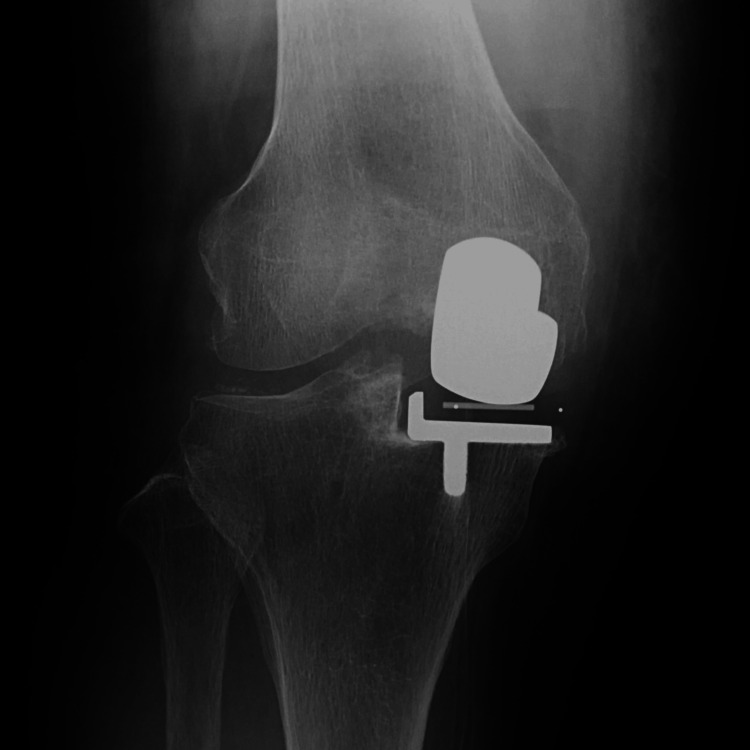
Radiography at two years postoperatively.

An 85-year-old woman (height of 144 cm, body weight of 44 kg, BMI of 21.2 kg/m²) underwent cementless Oxford UKA on the right side. Immediate postoperative radiographs showed no fracture line (Figure 13). The pin was placed outside the tibial mechanical axis. She was discharged without crutches two weeks postoperatively. At six weeks, there was no pain, tenderness, or swelling around the proximal tibia. An MRI showed similar fracture lines to Cases 1, 2, and 3 (Figure 14). No external fixation was performed, and bone union was observed at three months (Figure 15). There was no dislocation or loosening of components at two years postoperatively (Figure 16).

## Discussion

We reported four cases of tibial lateral condyle fractures that occurred after cementless Oxford UKA. Although tibial fracture is one of the most serious complications after Oxford UKA, all of the previously reported cases were of medial tibial fracture. Thus, this is the first report of lateral tibial plateau fractures as a postoperative complication of cementless Oxford UKA [6]. 

The rates of fractures of cemented Oxford UKA, cementless Oxford UKA, and hybrid Oxford UKA in our institution since 2014 were 1.1%, 4.4%, and 0.9%, respectively. These rates were comparable to previous reports [4,9]. Four tibial lateral condyle fractures occurred in cementless UKA, and other fractures were medial tibial plateau fractures. The reason for medial tibial plateau fractures is associated with surgical techniques, and there have been reports of medial tibial plateau fractures originating from the tibial cutting guide holding pinholes [6,7]. The lateral condyle fractures that we experienced in this study could be associated with surgical techniques and tibial medial condyle fractures, and we focused on the pinhole.

The fracture line originated from the intersection of the longitudinal and transverse cuts, passing the pinhole to the lateral tibial cortex. One of the causes of medial tibial plateau fractures was thought to be the pinholes [8]. In case the vertical cut is too deep, or the pinhole is located too far inside, there can be stress concentration at and around the pinhole [8]. In our hospital, to avoid this stress concentration, only a lateral pinhole is used when the tibial cutting guide is fixed. Additionally, a pin with a head is used for strong fixation. Further, to prevent the vertical cut from becoming too deep after finishing the horizontal cut, a thin metal plate is inserted in this area. Despite these preventive measures in this technique, fractures still occur. The lateral pinholes were placed outside the tibial mechanical axis in three of the four cases. The direction of the lateral bony cortex of the tibia is relatively close to the anteroposterior direction of the tibia [10]. In cases wherein the pinhole is too close to the cortical bone, it may be a cause of a decrease in bone strength. Moreover, if the transverse cut is excessive, it can become too close to the pinhole, which may be a cause of fractures.

Lateral tibial fractures after cementless Oxford UKA in our experience acquired good clinical results without the need for surgical intervention. Furthermore, this fracture did not pass through the implant in our patients. Prevention of medial fractures should be prioritized, and changing the order of osteotomies or the method of using pinholes is not necessary. However, the lateral pin should not be placed too far outside of the tibial axis. We have taken care not to place the pin outside the center of the tibial tuberosity. Until recently, we drove the pin in directly to improve fixation. However, to allow for some loss of fixation, our current policy is to insert the pin after first making a hole. 

In each of these four cases, tibial lateral condyle fractures were diagnosed by postoperative MRI. In our institution, all patients undergo MRI at six weeks postoperatively, which we think is useful for the diagnosis of occult medial tibial plateau fractures and loosening of components [11]. MRI is useful not only for these fractures but also for tibial lateral condyle fractures, which are less symptomatic.

## Conclusions

We reported four cases of postoperative tibial lateral condyle fractures after cementless Oxford UKA. All cases had slight or no symptoms and successfully healed without surgical intervention.

The prevention of medial tibial condyle fracture should be a priority, but the avoidance of too lateral placement of the tibial saw guide and careful pin insertion can avoid this complication.
